# Assessing intrinsic capacity in Taiwan: Initial psychometric properties of the Integrated Care for Older People Screening Tool for Taiwanese (ICOPES-TW)

**DOI:** 10.1186/s12877-024-05071-5

**Published:** 2024-05-31

**Authors:** Hui-Chen Su, Chieh-hsiu Liu, Hung-Yu Chen, Yi-Lin Wu, Mark D. Griffiths, Chung-Yi Li, Wen-Hsuan Hou, Chung-Ying Lin, Yi-Ching Yang

**Affiliations:** 1 Department of Neurology, National Cheng-Kung University Hospital, College of Medicine, National Cheng Kung University, Tainan, Taiwan; 2 Department of Family Medicine, Taoyuan General Hospital, Ministry of Health and Welfare, Taoyuan, Taiwan; 3 School of Medicine, National Tsing Hua University, Hsinchu, Taiwan; 4 Department of Family Medicine, National Cheng Kung University Hospital, College of Medicine, National Cheng Kung University, No. 1, University Rd, Tainan, 701401 Taiwan; 5 Department of Nursing, National Cheng Kung University Hospital, Tainan, Taiwan; 6 International Gaming Research Unit, Psychology Department, Nottingham Trent University, Nottingham, UK; 7 Department of Public Health, College of Medicine, National Cheng Kung University, Tainan, Taiwan; 8 Department of Public Health, College of Public Health, China Medical University, Taichung, Taiwan; 9 Department of Healthcare Administration, College of Medical and Health Science, Asia University, Taichung, Taiwan; 10 Department of Physical Medicine and Rehabilitation, Taipei Medical University Hospital, Department of Physcial Medicine and Rehabilitation, School of Medicine, College of Medicine, Taipei Medical University, Taipei, Taiwan; 11 Department of Geriatrics and Gerontology, National Cheng Kung University Hospital, National Cheng Kung University, Tainan, Taiwan; 12 Institute of Allied Health Sciences, College of Medicine, National Cheng Kung University, No. 1, University Rd, Tainan, 701401 Taiwan; 13 Faculty of Health and Life Sciences, INTI International University, Nilai, Malaysia; 14 Department of Occupational Therapy, College of Medicine, National Cheng Kung University, Tainan, Taiwan; 15 Biostatistics Consulting Center, National Cheng Kung University Hospital, College of Medicine, National Cheng Kung University, Tainan, Taiwan

**Keywords:** Aging, Frailty, Intrinsic capacity, Older people, Psychometrics, Quality of life

## Abstract

**Background:**

The World Health Organization (WHO) proposed the concept of intrinsic capacity (comprising composite physical and mental capacity) which aligns with their concepts of healthy aging and functional ability. Consequently, the WHO promotes the Integrated Care for Older People (ICOPE) framework as guidance for geriatric care. Consequently, each government should have a screening tool corresponding to ICOPE framework to promote geriatric care. The present study examined the initial psychometric properties of the Taiwan version of ICOPE (i.e., ICOPES-TW).

**Methods:**

Older people (*n* = 1235; mean age = 72.63 years; 634 females [51.3%]) were approached by well-trained interviewers for participation. A number of measures were administered including the ICOPES-TW, WHOQOL-AGE (assessing quality of life [QoL]), Clinical Frailty Scale (assessing frailty), Barthel Index (assessing basic activity of daily living [BADL]), and Lawton Instrumental Activities of Daily Living Scale (assessing instrumental activity of daily living [IADL]).

**Results:**

The ICOPES-TW had a two-factor structure (body functionality [eigenvalue = 1.932] and life adaptation [eigenvalue = 1.170]) as indicated by the results of exploratory factor analysis. Internal consistency of the ICOPES-TW was low (Cronbach’s α = 0.55 [entire ICOPES-TW], 0.45 (body functionality factor), and 0.52 (life adaptation factor). ICOPES-TW scores were significantly (i) positively correlated with age (*r* = 0.321), IADL (*r* = 0.313), and frailty (*r* = 0.601), and (ii) negatively correlated with QoL (*r*=–0.447), and BADL (*r*=–0.447), with all *p*-values < 0.001.

**Conclusion:**

The ICOPES-TW could be a useful screening tool for healthcare providers to quickly evaluate intrinsic capacity for Taiwanese older people given that it has moderate to strong associations with age, BADL, IADL, QoL, and frailty.

## Introduction

Aged or super-aged societies are now becoming the norm for almost every country worldwide due to healthcare advancements. Moreover, the World Health Organization (WHO) reported that life expectancy had increased from 66.8 years in 2000 to 73.4 years in 2019 [1]. Subsequently, how to overcome and deal with the issues associated with aged (or super-aged) societies is important globally [2, 3]. More specifically, aging results in older people having higher opportunities to encounter life difficulties and daily activity problems because aging leads to function decline [4, 5]. In other words, older people are likely to have decline in their functional ability (i.e., their ability to interact with their living environment), and the decline in functional ability may hinder healthy aging (i.e., maintenance of functional ability enables positive wellbeing). Therefore, geriatric care and healthy aging are important topics for healthcare providers and researchers in this field to assist older people in maintaining quality of life (QoL), dignity, and health.

The importance of maintaining QoL, dignity, and health for older people has been widely acknowledged and the WHO has proposed healthy aging to convert the stereotype that aging is associated with frailty [2, 3]. Although evidence shows that older people are at risk of having chronic diseases, comorbidities, body function declines, frailty, and disability [6, 7], the WHO promotes a positive attitude toward aging. Therefore, the concept of a capacity-based approach such as healthy aging and intrinsic capacity has been proposed and promoted [2, 8].

Intrinsic capacity has been defined as “the composite of the physical and mental capacities of an individual” [9], and is considered to be an important factor helping older people be successful in their healthy aging. In addition to intrinsic capacity, both physical and mental health have been emphasized to achieve healthy ageing. Therefore, it is important to know that intrinsic capacity shares some similar features with mental and physical health, but it also has some differences. More specifically, some aspects of intrinsic capacity may overlap with physical and mental health such as the physical and mental capacities of an individual [11]. However, intrinsic capacity focuses more on an individual’s functional ability (e.g., whether an older person can see, hear, walk, think, and remember), while physical and mental health relate more to the disorders or diseases themselves (e.g., whether an older person has a physical disease such as hypertension or has dopamine abnormalities causing cognitive problems). Therefore, when older individuals possess good intrinsic capacity, they are viewed as having capacities leading to good physical and mental health via various human biological systems with satisfactory body functions. Therefore, intrinsic capacity is a multidimensional and broad concept involving different operational indicators for healthcare providers and researchers in the field to evaluate older people regarding a clinical consortium on healthy aging [10].

The multidimensional nature of intrinsic capacity includes five key domains: cognition, locomotion, vitality, sensory (including visual and hearing), and psychological [11]. Cognition comprises older people’s abilities in problem-solving, intelligence, and memory; locomotion comprises abilities in mobility, muscle strength, and balance; vitality comprises abilities in hormonal function, cardio-respiratory function, and energy metabolism; sensory abilities comprise vision and hearing ability in daily living; and psychological abilities comprise emotion and mood performance [11].

Because the importance of intrinsic capacity has been clearly stated by experts [10], the WHO proposed and promoted the Integrated Care for Older People (ICOPE) framework to be a guide for geriatric care [2]. However, ICOPE itself cannot be successful if lacking a proper instrument to help healthcare providers assess older people’s intrinsic capacity. Therefore, researchers in geriatric care have been keen to develop screening tools assessing intrinsic capacity for older people taking into account the ICOPE framework [8, 12–16]. However, to the best of the present authors’ knowledge, most of these screening tools were validated using several existing tools to retrospectively correspond to the ICOPE framework [12–14, 16]. For example, older people’s cognition may be assessed using Mini Mental State Examination (MMSE), and Montreal Cognitive Assessment (MoCA) [8, 16] among other cognition screening tools. Moreover, no consensus has been reached regarding how to standardize a screening tool assessing intrinsic capacity [14].

Given that there is no consensus as to which screening tool can assess ICOPE globally, each country should at least have a unified screening tool for use. When a country has a unified screening tool for screening intrinsic capacity among older people, geriatric care policy can be more efficiently implemented in that country. In this regard, the Taiwan government developed a screening tool assessing intrinsic capacity comprising the five domains (i.e., cognition, locomotion, vitality, sensory, and psychological) proposed by the WHO alongside two additional domains (medication and life goals) [17]. The two items (i.e., medication and life goals) were added to the ICOPES-TW for the following reasons: (i) medication information provides information to clinicians to help them evaluate the severity of physical conditions of older people; (ii) medication is also an important concept for geriatric care included in the Geriatric 4Ms model (**M**edication, **M**obility, **M**entation, and what **M**atters) proposed by the Institute for Healthcare Improvement [18]; and (iii) the Geriatric 4Ms model also emphasizes the importance of older people’s concerns (i.e., what **M**atters), and this guided the Taiwan government to include life goals as one of the domains in this screening tool assessing intrinsic capacity. The screening tool was then named as the Integrated Care for Older People Screening Tool for Taiwanese (ICOPES-TW).

The WHO developed the concept of intrinsic capacity to highlight the various capabilities of an older adult, including the composite of all physical and mental capacities. Moreover, the ICOPE is a framework also developed by the WHO with the purpose of optimizing health and well-being among older adults through a person-centered and coordinated care approach. In this regard, the concept of intrinsic capacity is closely linked to the ICOPE because ICOPE focuses on identifying and addressing specific vulnerabilities and capabilities among older adults. In other words, the ICOPE can be used to design tailored interventions for older adults through the understanding of their intrinsic capacity, and to promote their functional ability and overall well-being. Subsequently, the domains constructed in the ICOPES-TW encompass the multidimensional aspects of intrinsic capacity. Therefore, ICOPES-TW can be viewed as a tool developed based on the concept of intrinsic capacity.

However, no psychometric evaluation of the ICOPES-TW has been carried out. Therefore, the present study evaluated the psychometric properties of the ICOPES-TW using the data collected from older people from different settings (including community, inpatient wards, and outpatient clinics). Although the WHO proposes evaluating intrinsic capacity for community-residing older people, the concept of intrinsic capacity can also be applied to older people residing in different settings (e.g., the aforementioned inpatient wards and outpatient clinics) when such older people are not severely ill. In this regard, using ICOPES-TW to evaluate intrinsic capacity among older people in different settings would expand its utility in providing health information regarding older people for healthcare providers. Subsequently, this information could be used by healthcare providers in different settings to foster appropriate geriatric care programs. Therefore, the present study carried out reliability and validity testing for the ICOPES-TW through different types of psychometric evaluation in different settings.

The present study had an overall aim of examining the initial psychometric properties (including reliability and validity) of the ICOPES-TW. In addition to the overall aim, there were seven more specific reasons for present study. First, descriptive statistics were used to understand participants’ characteristics and ICOPES-TW item score distributions in the present study. Second, construct validity (using exploratory factor analysis [EFA]) was used to identify the structure of the ICOPES-TW because this could help healthcare providers understand what types of intrinsic capacity are assessed. Third, internal consistency (using Cronbach’s α) was used to help improve the understanding if ICOPES-TW subscales have internal coherence to each other. Fourth, concurrent validity (using Pearson correlation) was used to examine if the ICOPES-TW is associated with other health measures. Fifth, predictive validity (using receiver operating characteristic [ROC] curve) was used to examine if the ICOPES-TW could satisfactorily predict frailty and activity of daily living (ADL) dependency. Sixth, discriminant power (using analysis of variance [ANOVA] or independent t-tests) was used to examine if ICOPES-TW could distinguish older people in different subgroups, such as from different settings (i.e., community, outpatient, and inpatient). Although the WHO proposed intrinsic capacity for community-residing older people, the present study expanded the use of intrinsic capacity to older people across different settings as long as they were not severely ill. Lastly, regression models were used to examine how the ICOPES-TW associates with QoL and frailty.

## Methods

### Participants and data collection

After obtaining the approval from the National Cheng Kung University Hospital (NCKUH) Institutional of Review Board (IRB No.: A-ER-110-249), several interviewers were trained to interact and collect data from potential participants. This included explaining the research purpose, guiding participants to answer questionnaires, and observing and assessing the participants’ condition and performance. In total, there were 10 interviewers who helped in the data collection, and each of them attended a two-hour training workshop with an evaluation by some of research team to ensure their consistency and ability to conduct data collection. The inclusion criteria for eligible participants were (i) being aged above 50 years; (ii) being able to communicate using Mandarin Chinese or Taiwanese; and (iii) having the ability to provide consent for participation. The exclusion criteria were (i) not providing consent; (ii) having difficulties in understanding the questionnaires used; and (iii) being severely ill based on medical records. Although there were different sources for data collection (community, inpatient wards, and outpatient clinics), the inclusion and exclusion criteria were the same across for all the sources. Therefore, the present sample was viewed as healthy.

There were two target populations in the present study: one was older people who lived in Tainan City, and the other was the patient population of the NCKUH. Therefore, the data were collected from individuals (i) living in the community in Tainan City, (ii) in inpatient wards in the NCKUH, and (iii) in outpatient clinics in the NCKUH. To recruit the participants living in the community, trained interviewers visited community centers at the time of older adults’ annual health check-ups using convenience sampling. The interviewers then provided detailed information regarding the data collection procedure for those who were interested in participating in the study. To recruit individuals from inpatient and outpatient wards, several collaborating physicians helped in identifying eligible participants and provided the names to the interviewers. For inpatients, the interviewers visited potential participants in the wards, explained the study, and invited them to participate. For outpatients, the interviewers were provided with the times that these individuals would be visiting the outpatient clinic. The interviewers waited outside the outpatient clinic and approached the individuals after their appointments at the outpatient visit, explained the study, and invited them to participate. Moreover, there was no specific time (e.g., in admission or discharge) for the interviewers to recruit inpatients and outpatients. The participants were approached for participation only when the physicians evaluated them as being stable and eligible. Therefore, there was no impact as a result of hospitalization for the data collection. In all three types of recruitment, after agreeing to participate, the interviewer asked the person to sign a written informed consent and then conducted the interview to collect the data. The present study’s procedure followed and adhered to the declaration of the Helsinki with all participants providing written informed consent for participation.

### Sample size calculation

The present study used several methods to determine the required sample size. For the EFA, a rule-of-thumb estimation method was used. More specifically, an item-person ratio of 1:10 or a minimum sample size of 400 to 500 was used (depending upon which sample size calculation was larger) [19]. Because the ICOPES-TW contains seven subscales for EFA, the sample size should therefore contain 400 or more participants. Second, because concurrent validity was calculated using Pearson correlation, sample size was also calculated using Pearson correlation. With an *r*-value at 0.3, power at 0.8, type I error at 0.05, and a two-sided test, the required sample size was 84. Third, because ANOVA was used to examined discriminant power, ANOVA was used to calculate the required sample size. With a medium effect size (i.e., f = 0.25), power at 0.8, type I error at 0.05, and a two-sided test, the required sample size was 159.

### Measures

*Integrated Care for Older People Screening Tool for Taiwanese (ICOPES-TW).* The ICOPES-TW is an eight-subscale instrument used to assess intrinsic capacity in conjunction with the WHO ICOPE framework [2]. The ICOPES-TW was initially developed by a group of experts in Gerontology and Geriatrics after reviewing the WHO ICOPE framework documents and other relevant studies in promoting ICOPE. The group of experts comprised individuals from the Health Promotion Administration, Ministry of Health and Welfare in Taiwan, and who were responsible for healthy aging promotion. After reviewing the literature regarding prior studies on intrinsic capacity [9–12] and the concepts of WHO ICOPE framework [2], the group of experts used their expertise and experiences to generate the initial version of the ICOPES-TW. The ICOPES-TW was developed as a screening tool comprising quick assessments of all the intrinsic capacity domains.

More specifically, every domain of intrinsic capacity (including cognition, locomotion, vitality, visual, hearing, and psychological) defined by the WHO is included as a subscale in the ICOPES-TW. Moreover, the ICOPES-TW contains two additional subscales (medication and life goals). However, given that the life goals item in the ICOPES-TW is an open answer question, this subscale was not used in the present study to screen intrinsic capacity among older people. Regarding the remaining seven subscales, the cognition subscale included three items (time orientation; location orientation; three-item recall memory); the locomotion subscale included one item (mobility); the vitality subscale included two items (weight loss over 3 kg; loss of appetite); the visual subscale included one item (difficulty in watching); the hearing subscale included one item (ability in repeating the numbers 6, 1, and 9); the psychological subscale included two items (feeling bothersome; reducing engagements in activities); and the medication subscale included three items (taking ten or more different medications; taking painkillers or sleeping tablets; change in balance, sleepiness, dizziness, low blood hypertension, or mouth dry due to medication taken). All the subscales were then converted into 0 (no problems) or 1 (having problems), with higher ICOPES-TW scores indicating poorer intrinsic capacity. Apart from the cognition and hearing subscales, which were assessed and rated by the interviewers, all other subscales were self-reported by the participants.

*WHOQOL-AGE.* The WHOQOL-AGE is a 13-item self-report instrument used to assess quality of life (QoL) among older people through the lens of older people’s daily living [20, 21]. All items were rated using a five-point Likert-type scale with a higher score indicating better QoL (individual item scores range from 0 to 4 with the 13-item WHOQOL-AGE total score ranging from 0 to 52). The WHOQOL-AGE has been validated in Chinese among Taiwanese older people with promising psychometric properties [22]. The internal consistency of the WHOQOL-AGE in the present study’s sample was excellent (α = 0.931).

*Clinical Frailty Scale (CFS).* The CFS comprises one item administered by an interviewer to assess the frailty of an older individual. The CFS item included nine responses from 1 (*very fit*) to 9 (*terminally ill*). Therefore, a higher CFS score indicates higher frailty among older people [23, 24]. The CFS has been validated in Chinese among Taiwanese older people with promising psychometric properties [25].

*Barthel Index (BI).* The BI is a 10-item self-report instrument that assesses older people’s basic ADL (BADL). All items are rated using a scale with either two scores (0 and 5), three scores (0, 5, and 10), or four scores (0, 5, 10, and 15) with a higher score indicating better BADL. All item scores were summed to indicate the overall performance concerning BADL ranging from 0 (indicating totally dependent) to 100 (indicating totally independent) [26]. The BI has been validated in Chinese among Taiwanese older people with promising psychometric properties [27]. The internal consistency of the BI in the present study’s sample was excellent (α = 0.930).

*Lawton Instrumental Activities of Daily Living Scale (Lawton IADLS).* The Lawton IADLS is an eight-item self-report instrument that assesses older people’s instrumental activity of daily living (IADL). All items were rated using a dichotomous scale in dependence (scoring 1) or independence (scoring 0) with higher scores indicating poorer IADL. All item scores were summed to indicate the overall performance of IADL ranging from 0 (indicating totally independent) to 8 (indicating totally dependent) [28]. The Lawton IADLS has been validated in Chinese among Taiwanese older people with promising psychometric properties [29, 30]. The internal consistency of the Lawton IADLS in the present study’s sample was excellent (α = 0.954).

*Demographics.* Information concerning several demographic variables was collected. This included participant age (answered in years), sex (answered male or female), educational level (answered as primary or below, junior or senior high, or college or above), marital status (answered married, widowed or other), and living status (answered living alone or not living alone).

### Data analysis

Descriptive statistics were used to summarize the participants’ demographic characteristics and their scores on the measures of WHOQOL-AGE, CFS, BI, and Lawton IADLS. Moreover, ceiling and floor effects of the ICOPES-TW were calculated using the percentages at the best (i.e., score 0) and the worst (i.e., score 7) scores of the ICOPES-TW. Because the ICOPES-TW has never been previously explored for its potential factor structure, exploratory factor analysis (EFA) using the principal axis factoring extraction method was used to identify the potential factors comprising the ICOPES-TW. Before performing the EFA, Kaiser-Keyer-Olkin (KMO) over 0.6 and a significant Bartlett’s tests were used to evaluate if the sample was adequate and suitable for the EFA [31]. In the EFA, ICOPES-TW subscale scores were used for the extraction and the number of factors was decided using multi-methods, including the Kaiser’s rule (i.e., eigenvalue > 1) [32] and parallel analysis. In the parallel analysis, 100 Monte Carlo simulations were used to calculate the eigenvalues. When the 95% confidence interval (CI) upper limit eigenvalue derived from the simulations for a factor is greater than the eigenvalue calculated from the present dataset, that factor is considered to be true [33]. Moreover, a factor loading > 0.3 is considered to be adequate for the EFA findings [31]. After deciding the factor structure of the ICOPES-TW, internal consistency together with corrected item-total correlation were examined for the entire ICOPES-TW and its potential factors.

The concurrent validity of the ICOPES-TW was assessed using the Pearson correlations between the ICOPES-TW scores (total score and potential factor scores) and the following variables: age (older age was expected to be correlated with higher ICOPES-TW score), BI (higher BI score was expected to be correlated with lower ICOPES-TW score), Lawton IADLS (higher Lawton IADLS score was expected to be correlated with higher ICOPES-TW score), WHOQOL-AGE (higher WHOQOL-AGE score was expected to be correlated with lower ICOPES-TW score), and CFS (higher CFS score was expected to be correlated with higher ICOPES-TW score). These variables were used because prior evidence has indicated that intrinsic capacity is correlated with all of them. More specifically, poor intrinsic capacity has been correlated with (i) older age, (ii) poorer BADL [12], lower QoL [34], and more severe frailty [35].

After performing concurrent validity, the CFS and BI were further used to examine the predictive validity of the ICOPES-TW. Firstly, the CFS score was recoded as moderate frailty to terminally ill (scores from 6 to 9) and healthy to mild frailty (scores from 0 to 5). BI score was recoded as moderate to total dependency (scores from 0 to 60) and independent to mild dependency (scores from 61 to 100). Following this, the ROC curve was used to examine if ICOPES-TW score could predict moderate frailty and moderate dependency in ADL. The area under ROC curve (AUC) was used for deciding the predictive validity, of which AUC > 0.7 indicates good predictive validity.

Independent *t*-tests and analyses of variance (ANOVAs) with Bonferroni adjustment were then used to evaluate whether the ICOPES-TW score performed differently between the following demographic variables: sex (males vs. females), living status (alone vs. not alone), educational level (primary school or below, high school, and college or above), and marital status (married, widowed, and other). ANOVA with Bonferroni adjustment was additionally used to examine the discriminant power of the ICOPES-TW to evaluate whether the ICOPES-TW could significantly distinguish older people from different settings (i.e., community, outpatient, and inpatient).

Because QoL and frailty are considered to be important outcomes among older people [36, 37], their correlations with the ICOPES-TW were further examined. More specifically, several regression models were constructed using the following criteria: WHOQOL-AGE and CFS were treated as dependent variables each with the same set of independent variables comprising age, sex (reference group: female), educational level (reference group: primary school or below), marital status (reference group: married), living status (reference group: living alone), BI, Lawton IADLS, and ICOPES-TW. All the statistical analyses were performed using SPSS 17.0 (SPSS Inc., Chicago, IL).

## Results

### Participant characteristics, ICOPES-TW item score distribution, and statistical power

The statistical power was over 0.95 for both the Pearson correlation and ANOVA findings used in the present study. The mean age of the participants (*N* = 1235) was 72.63 years (SD = 7.19) ranging between 59 and 97 years. The gender distribution was relatively balanced (634 females; 51.3%) with most participants being currently married (*n* = 886; 71.7%). Slightly over one-fifth of the participants had completed educational level at college or above (*n* = 250; 20.2%). Most of the participants were not living alone (*n* = 1098; 88.9%). Most of the participants were recruited via outpatient clinics (*n* = 691; 56.0%), followed by those from the community (*n* = 421; 34.1%) and inpatient wards (*n* = 123; 9.9%). There were slightly high ceiling effects (35.1%) but negligible floor effects (0.3%) for the ICOPES-TW. However, most participants had ICOPES-TW scores at 2 or below (80.7%), indicating the composition of many healthy older adults in the present sample. In addition, the quartiles of the ICOPES-TW were 0.00 (first quartile), 1.00 (second quartile), and 2.00 (third quartile). Table 1 further reports the participants’ performance concerning BADL, IADL, QoL, and frailty. Also, the information for the three groups is reported separately in Table 1.

**Table 1:** Participants’ characteristics (*N* = 1235)

		M (SD) or *n* (%)		
	All sample (*N* = 1235)	Community (*n* = 421)	Outpatient (*n* = 691)	Inpatient (*n* = 123)
Age (year)	72.63 (7.19)	72.22 (5.75)	72.56 (7.63)	74.37 (8.74)
Sex				
Women	634 (51.3)	232 (55.1)	344 (49.8)	58 (47.2)
Men	601 (48.7)	189 (44.9)	347 (50.2)	65 (52.8)
Educational level				
Primary school or below	491 (39.8)	171 (40.6)	258 (37.3)	62 (50.4)
High school	477 (38.6)	179 (42.5)	253 (36.6)	45 (36.6)
College or above	250 (20.2)	61 (14.5)	176 (25.5)	13 (10.6)
Missing data	17 (1.4)	10 (2.4)	4 (0.6)	3 (2.4)
Marital status				
Married	886 (71.7)	321 (76.2)	490 (70.9)	75 (61.0)
Widowed	254 (20.6)	78 (18.5)	147 (21.3)	29 (23.6)
Other	95 (7.7)	22 (5.2)	54 (7.8)	19 (15.4)
Living status				
Living alone	136 (11.0)	53 (12.6)	68 (9.8)	15 (12.2)
Not living alone	1098 (88.9)	368 (87.4)	623 (90.2)	107 (87.0)
Missing data	1 (0.1)	0 (0.0)	0 (0.0)	1 (0.8)
ICOPES-TW score (Range: 0–7)	1.36 (1.43)	0.98 (1.11)	1.28 (1.36)	3.10 (1.55)
Score 0 (i.e., ceiling effect)	433 (35.1)	175 (41.6)	255 (36.9)	3 (2.4)
Score 1	341 (27.6)	141 (33.5)	181 (26.2)	19 (15.4)
Score 2	222 (18.0)	63 (15.0)	139 (20.1)	20 (16.3)
Score 3	126 (10.2)	27 (6.4)	64 (9.3)	35 (28.5)
Score 4	67 (5.4)	11 (2.6)	33 (4.8)	23 (18.7)
Score 5	31 (2.5)	3 (0.7)	12 (1.7)	16 (13.0)
Score 6	11 (0.9)	1 (0.2)	6 (0.9)	4 (3.3)
Score 7 (i.e., floor effect)	4 (0.3)	0 (0.0)	1 (0.1)	3 (2.4)
BADL score (Range: 0-100)	95.96 (13.64)	99.74 (1.52)	96.51 (11.15)	80.00 (29.36)
IADL score (Range: 1–9)	1.14 (0.59)	1.03 (0.21)	1.15 (0.67)	1.43 (0.85)
QoL score (Range: 8–52)	34.29 (6.53)	36.48 (5.26)	34.02 (6.51)	28.14 (6.50)
Frailty score (Range: 1–9)	2.53 (1.30)	0.98 (1.11)	2.65 (1.17)	4.22 (1.58)

ICOPES-TW = Integrated Care for Older People Screening Tool for Taiwanese; BADL = basic activity of daily living (assessed using Barthel Index); IADL = instrumental activity of daily living (assessed using Lawton Instrumental Activities Daily Living Scale); QoL = quality of life (assessed using WHOQOL-AGE); Frailty assessed using Clinical Frailty Scale

Higher scores in ICOPES-TW indicate poorer intrinsic capacity; higher scores in Barthel Index indicate better BADL; higher scores in Lawton Instrumental Activities Daily Living Scale indicate poorer IADL; higher scores in WHOQOL-AGE indicate better QoL; higher scores in CFS indicate higher frailty

### Construct validity using EFA

The KMO was 0.69 and the Bartlett’s test was significant (χ^2^ = 538.99, *df* = 21; *p* < 0.001), indicating the sample adequacy and suitability for the EFA. Regarding the psychometric properties of the ICOPES-TW, both Kaiser’s rule and parallel analysis results suggested there were two factors for the seven subscales (see Table 2 for parallel analysis results). Factor 1 (named ‘body functionality’; eigenvalue = 1.932) explained 27.69% of the variance, with cognition, locomotion (i.e., individuals’ mobility), visual, and hearing subscales; Factor 2 (named ‘life adaptation’; eigenvalue = 1.170) explained 16.71% of the variance, with vitality, psychological, and medication subscales (medication was considered as life adaptation because individuals have to adjust their living schedule to take various medications) (Table 3). Although the visual subscale had a low factor loading (i.e., < 0.3), it was retained because visual ability is a key ability proposed by the WHO for intrinsic capacity.

**Table 2:** Results of parallel analysis

Factor #		Eigenvalue	
	From raw data	Mean of simulations	95% upper limit confidence interval of simulations
1	1.94	1.10	1.14
2	1.17	1.06	1.08
3	0.94	1.03	1.05
4	0.82	1.00	1.02
5	0.77	0.97	0.99
6	0.73	0.94	0.96
7	0.63	0.90	0.93

**Table 3:** Subscale properties of the Integrated Care for Older People Screening Tool for Taiwanese (ICOPES-TW) (*N* = 1235)

Factor Subscale	*n* (%) having a problem	Factor loading	Corrected item-total correlation^a^	Corrected item-total correlation^b^
**Factor 1 (Body functionality)**				
Cognition	284 (23.0)	0.53	0.23	0.27
Locomotion	341 (27.6)	0.42	0.38	0.30
Hearing	292 (23.6)	0.42	0.28	0.28
Visual	229 (18.5)	0.24	0.18	0.17
**Factor 2 (Life adaptation)**				
Vitality	205 (16.6)	0.63	0.29	0.35
Psychological	211 (17.1)	0.50	0.35	0.36
Medication	118 (9.6)	0.40	0.24	0.29

^a^ Corrected item-total correlation using all subscales (Cronbach’s α = 0.55)

^b^ Corrected item-total correlation using factor subscales (Cronbach’s α = 0.45 for Factor 1 [Body functionality]; 0.52 for Factor 2 [Life adaptation])

Factor loadings were derived using exploratory factor analysis with the principal axis factoring extraction method

*Note*The subscale scoring was: 0 (no problems for all the items assessed in that specific subscale) or 1 (any problems reported for the items assessed in that specific subscale)

### Internal consistency using Cronbach’s α

Internal consistency of these subscales was somewhat low: Cronbach’s α = 0.55 (entire ICOPES-TW), 0.45 (for body functionality factor), and 0.52 (for life adaptation factor). Moreover, corrected item-total correlations among the subscales were relatively low from 0.17 to 0.38 (Table 3).

### Concurrent validity using Pearson correlation

The ICOPES-TW total score was significantly correlated with all external criteria at a moderate magnitude: absolute *r* = 0.313 to 0.601 (all *p*-values < 0.001). The ICOPES-TW body functionality factor was also significantly correlated with all external criteria at a moderate magnitude: absolute *r* = 0.305 to 0.515 (all *p*-values < 0.001). The ICOPES-TW life adaptation factor was significantly correlated with all external criteria with a weak to moderate magnitude: absolute *r* = 0.078 to 0.434 (all *p*-values < 0.001).

Moreover, the ICOPES-TW body functionality factor had stronger correlations with BADL, IADL, and frailty than the ICOPES-TW life adaptation factor. The ICOPES-TW life adaptation factor had stronger correlations with QoL than the ICOPES-TW body functionality factor (Table 4).

**Table 4:** Concurrent validity of Integrated Care for Older People Screening Tool for Taiwanese (ICOPES-TW)

		*r*	
	1	2	3
1. ICOPES-TW (total)	--		
2. ICOPES-TW (BF)	0.713	--	
3. ICOPES-TW (LA)	0.863	0.261	--
4. Age	0.321	0.386	0.078
5. BADL	-0.447	-0.423	-0.268
6. IADL	0.313	0.305	0.174
7. QoL	-0.447	-0.315	-0.417
8. Frailty	0.601	0.515	0.434

*Note* All *p*-values < 0.001

BF = body functionality; LA = life adaptation; BADL = basic activity of daily living (assessed using Barthel Index); IADL = instrumental activity of daily living (assessed using Lawton Instrumental Activities Daily Living Scale); QoL = quality of life (assessed using WHOQOL-AGE); Frailty was assessed using Clinical Frailty Scale

Higher scores in ICOPES-TW indicate poor intrinsic capacity; higher scores in Barthel Index indicate better BADL; higher scores in Lawton Instrumental Activities Daily Living Scale indicate poorer IADL; higher scores in WHOQOL-AGE indicate better QoL; higher scores in CFS indicate higher frailty

### Predictive validity using the ROC curve

Table 5 shows the predictive validity of the ICOPES-TW. More specifically, the ICOPES-TW total score had satisfactory AUC in predicting moderate frailty (0.894) and moderate dependency in ADL (0.892). Moreover, the optimal cutoff score for identifying at-risk frailty and ADL dependency was scoring 1 in the ICOPES-TW total score. With regard to the two factors of the ICOPES-TW, both had acceptable AUC: the body functionality factor had an AUC of 0.875 for identifying at-risk frailty and 0.725 for ADL dependency. The life adaptation factor had an AUC of 0.725 for identifying at-risk frailty and 0.729 for ADL dependency. For both factors, the cutoff scores also scored 1.

**Table 5:** Predictive validity of Integrated Care for Older People Screening Tool for Taiwanese (ICOPES-TW) using receiver operating characteristic (ROC) curve

	Clinical Frailty Scale			Barthel Index	
	Sensitivity	1 - Specificity		Sensitivity	1 - Specificity
**ICOPES-TW (total)**	**AUC = 0.894**			**AUC = 0.892**	
Score 1	1.000	0.635		1.000	0.636
Score 2	0.918	0.351		0.915	0.352
Score 3	0.857	0.166		0.830	0.168
**ICOPES-TW (BF)**	**AUC = 0.875**			**AUC = 0.889**	
Score 1	1.000	0.548		1.000	0.549
Score 2	0.816	0.222		0.851	0.221
Score 3	0.551	0.073		0.596	0.072
**ICOPES-TW (LA)**	**AUC = 0.725**			**AUC = 0.729**	
Score 1	0.673	0.283		0.702	0.283
Score 2	0.429	0.097		0.362	0.100
Score 3	0.122	0.019		0.128	0.019

AUC = area under ROC curve; BF = body functionality; LA = life adaptation

Clinical Frailty Scale recoded as 0 (scores 1–5; healthy to mild frailty) and 1 (scores 6–9; moderate frailty to terminally ill)

Barthel Index recoded as 0 (scores 61–100; mild dependency to independency) and 1 (scores 0–60; moderate dependency to total dependency)

Higher scores in ICOPES-TW indicate poor intrinsic capacity

### Discriminant power using ANOVA or independent t-tests

Significant differences in the ICOPES-TW total score were found between sex groups (mean = 1.45 [females] vs. 1.27 [males]; *p* = 0.024), educational level (mean = 1.70 [primary school or below], 1.21 [high school], and 0.98 [college or above]; *p* < 0.001), and marital status (mean = 1.25 [married], 1.65 [widowed], and 1.60 [others]; *p* < 0.001) but not living status (mean = 1.48 [alone] vs. 1.34 [not alone]; *p* = 0.272). Moreover, significant differences in the ICOPES-TW body functionality factor score were found between education (*p* < 0.001) and marital status (*p* < 0.001) but not in sex (*p* = 0.082) and living status (*p* = 0.636). A significant difference in the ICOPES-TW life adaptation factor score was found between marital status (*p* = 0.006) but not in sex, living status, and educational level (*p*-values = 0.058 to 0.176) (Table 6). Moreover, the discriminant power of the ICOPES-TW was supported as it was able to significantly distinguish older people from different settings (Table 7).

**Table 6:** Comparing Integrated Care for Older People Screening Tool for Taiwanese (ICOPES-TW) between sex, living status, educational level, and marital status

	ICOPES-TW (total)		ICOPES-TW (BF)		ICOPES-TW (LA)	
	M (SD)	*t* or F (*p*)	M (SD)	*t* or F (*p*)	M (SD)	*t* or F (*p*)
Sex		2.26 (0.024)		1.74 (0.082)		1.90 (0.058)
Females (*n* = 634)	1.45 (1.48)		0.98 (1.05)		0.47 (0.78)	
Males (*n* = 601)	1.27 (1.37)		0.88 (1.02)		0.39 (0.71)	
Living status		1.10 (0.272)		0.47 (0.636)		1.35 (0.176)
Living alone (*n* = 136)	1.48 (1.32)		0.96 (0.95)		0.51 (0.80)	
Not living alone (*n* = 1098)	1.34 (1.44)		0.92 (1.05)		0.42 (0.74)	
Educational level^a^		26.03 (< 0.001)		39.68 (< 0.001)		2.04 (0.130)
Primary school or below (*n* = 491)	1.70 (1.54)		1.24 (1.15)		0.46 (0.79)	
High school (*n* = 477)	1.21 (1.34)		0.77 (0.90)		0.44 (0.76)	
College or above (*n* = 250)	0.98 (1.22)		0.64 (0.90)		0.35 (0.64)	
Marital status^b^		9.23 (< 0.001)		12.62 (< 0.001)		5.09 (0.006)
Married (*n* = 886)	1.25 (1.38)		0.85 (0.98)		0.41 (0.73)	
Widowed (*n* = 254)	1.65 (1.50)		1.21 (1.17)		0.44 (0.72)	
Other (*n* = 95)	1.60 (1.53)		0.94 (1.01)		0.66 (0.91)	

^a^ Bonferroni adjustment showed that college or above group had significant differences from primary or below group in ICOPES-TW total score and BF score

^b^ Bonferroni adjustment showed that the married group had significant differences from the widowed group in ICOPES-TW total score and BF score; the married group had significant differences from the ‘other’ group in ICOPES-LA score

BF = body functionality; LA = life adaptation

**Table 7:** Discriminant power of the Integrated Care for Older People Screening Tool for Taiwanese (ICOPES-TW)

		M (SD)		F-value (*p*-value)	Bonferroni adjustment
	1. Community (*n* = 421)	2. Outpatient (*n* = 691)	3. Inpatient (*n* = 123)		
ICOPES-TW (total)	0.98 (1.11)	1.28 (1.36)	3.10 (1.55)	129.09 (< 0.001)	3 > 2 > 1
ICOPES-TW (BF)	0.83 (0.97)	0.80 (0.95)	1.98 (1.12)	80.01 (< 0.001)	3 > 2 > 1
ICOPES-TW (LA)	0.15 (0.41)	0.48 (0.76)	1.11 (1.03)	94.48 (< 0.001)	3 > 2 = 1

BF = body functionality; LA = life adaptation; Higher scores indicate poorer conditions in ICOPES-TW

### Relationships between ICOPES-TW, QoL, and frailty using regression models

Regression models additionally showed that the ICOPES-TW had strong correlations with QoL and frailty after controlling for age, sex, educational level, marital status, living status, BADL, and IADL (Table 8). More specifically, the standardized coefficient (β) of the ICOPES-TW total score was − 0.35 (*p* < 0.001) when explaining QoL (changed R^2^ = 0.088); 0.30 (*p* < 0.001; when explaining frailty (changed R^2^ = 0.113). Similarly, the β of the ICOPES-TW body functionality was − 0.12 (*p* < 0.001) for QoL and 0.19 (*p* < 0.001) for frailty (changed R^2^ = 0.083); the β of the ICOPES-TW life adaptation was − 0.32 (*p* < 0.001) for QoL and 0.24 (*p* < 0.001) for frailty (changed R^2^ = 0.083) (Table 8).

**Table 8:** Regression model of Integrated Care for Older People Screening Tool for Taiwanese (ICOPES-TW) on quality of life (QoL) and frailty

	Unstandardized coefficient (SE)/Standardized coefficient (*p*-value)			
	Model 1 (DV: QoL)	Model 2 (DV: QoL)	Model 3 (DV: Frailty)	Model 4 (DV: Frailty)
Age	0.08 (0.03)/ 0.08 (0.003)**	0.05 (0.03)/ 0.06 (0.047)*	0.02 (0.004)/ 0.10 (< 0.001)***	0.02 (0.004)/ 0.11 (< 0.001)***
Sex (Ref: females)	-0.04 (0.35)/ -0.003 (0.91)	-0.18 (0.35)/ -0.01 (0.61)	0.02 (0.06)/ 0.01 (0.68)	0.03 (0.06)/ 0.01 (0.53)
Education (Ref: ≦primary school)				
High school	-0.53 (0.66)/ -0.04 (0.42)	-0.01 (0.65)/ -0.001 (0.98)	-0.13 (0.10)/ -0.05 (0.20)	-0.17 (0.10)/ -0.07 (0.09)
≧College	0.24 (0.69)/ 0.02 (0.73)	0.88 (0.69)/ 0.07 (0.20)	-0.28 (0.11)/ -0.11 (0.008)**	-0.34 (0.11)/ -0.13 (0.002)**
Marital status (Ref: married)				
Widowed	-0.56 (0.47)/ -0.04 (0.24)	-0.69 (0.46)/ -0.04 (0.14)	0.08 (0.07)/ 0.02 (0.28)	0.09 (0.07)/ 0.03 (0.20)
Others	-1.44 (0.68)/ -0.06 (0.03)*	-1.28 (0.67)/ -0.05 (0.06)	0.23 (0.10)/ 0.05 (0.03)*	0.22 (0.10)/ 0.04 (0.04)*
Living status (Ref: living alone)	-0.30 (0.59)/ -0.02 (0.61)	-0.40 (0.58)/ -0.02 (0.49)	0.06 (0.07)/ 0.02 (0.40)	0.08 (0.07)/ 0.02 (0.30)
BADL	0.09 (0.02)/ 0.19 (< 0.001)***	0.10 (0.02)/ 0.21 (< 0.001)***	-0.04 (0.002)/ -0.42 (< 0.001)***	-0.04 (0.002)/ -0.42 (< 0.001)***
IADL	-0.92 (0.33)/ -0.08 (0.005)**	-0.94 (0.32)/ -0.09 (0.004)**	0.11 (0.05)/ 0.05 (0.03)*	0.11 (0.05)/ 0.05 (0.03)*
ICOPES-TW (total)	-1.57 (0.13)/ -0.35 (< 0.001)***	--	0.30 (0.02)/ 0.34 (< 0.001)***	--
ICOPES-TW (BF)	--	-0.72 (0.19)/ -0.12 (< 0.001)***	--	0.23 (0.03)/ 0.19 (< 0.001)***
ICOPES-TW (LA)	--	-2.76 (0.23)/ -0.32 (< 0.001)***	--	0.41 (0.04)/ 0.24 (< 0.001)***
	**Fit and diagnostic statistics**			
R^2^ (adjusted R^2^)	0.259 (0.253)	0.284 (0.278)	0.548 (0.544)	0.553 (0.549)
Changed R^2^ (changed adjusted R^2^)^a^	0.088 (0.088)	0.113 (0.113)	0.083 (0.083)	0.088 (0.088)
F (p)	40.56 (< 0.001) ***	41.83 (< 0.001) ***	141.30 (< 0.001) ***	130.96 (< 0.001) ***
Variance inflation factor	1.153–4.358	1.128–4.451	1.151–4.314	1.127–4.399

*p < 0.05; **p < 0.01; ***p < 0.001; DV = dependent variable; BF = body functionality; LA = life adaptation; BADL = basic activity of daily living (assessed using Barthel Index); IADL = instrumental activity of daily living (assessed using Lawton Instrumental Activities Daily Living Scale); QoL = quality of life (assessed using WHOQOL-AGE); Frailty was assessed using Clinical Frailty Scale. Higher scores indicate poorer conditions in ICOPES-TW, Lawton Instrumental Activities Daily Living Scale, and Clinical Frailty Scale; better conditions in Barthel Index and WHOQOL-AGE

^a^ Changed R^2^ (changed adjusted R^2^) indicates the increase of R^2^ (adjusted R^2^) for the ICOPES-TW (total) or ICOPES-TW (BF) together with ICOPES-TW (LA)

## Discussion

Using data collected from 1235 older people in Taiwan, the present study evaluated the initial psychometric properties of the newly developed ICOPES-TW. A ceiling effect was observed for the ICOPES-TW and is most likely explained by the sample composition (i.e., less than 10% of the participants were inpatients, indicating that the majority of the participants were relatively healthy). Using an older adult sample from southern Taiwan, the results indicated that the ICOPES-TW comprised a two-factor structure (body functionality and life adaptation) in assessing older people’s intrinsic capacity. However, internal consistency of the ICOPES-TW was low for the ICOPES-TW total score and its two factor scores. In addition, although the two items added to the ICOPES-TW (i.e., medication and life goals) differentiate the ICOPES-TW from the original ICOPE, additional psychometric evidence is required to support their inclusion in assessing intrinsic capacity. More specifically, the item assessing life goals requires an open-ended response and its psychometric properties cannot be verified. Therefore, the present study only provided initial psychometric evidence for the ICOPES-TW and future studies are needed to assess the two additional items (i.e., medication and life goals). However, the ICOPES-TW had moderate correlations with several external criteria including age, ADL, QoL, and frailty, indicating its ability in assessing intrinsic capacity among older people. Moreover, older people with a higher level of education had better ICOPES-TW scores than those with a lower level of education.

The internal consistency of the ICOPES-TW was low given that its Cronbach α values were 0.4 to 0.5, much lower than the acceptable standard of 0.7 [38]. However, the ICOPES-TW is a screening measure with multidimensionality. Therefore, the low internal consistency could be due to this. More specifically, screening tools are not expected to thoroughly and comprehensively assess a latent construct [39]. Instead, the main purpose of a screening tool is to quickly detect potential health risks for an individual [40]. Based on these observations, screening tools may not have sufficient items to assess a similar concept and result in relatively low internal consistency. Moreover, the ICOPES-TW was designed to cover all aspects of intrinsic capacity among older people. Therefore, the diversity among these subscales could be large. The diversity among the subscales together with the use of too few items are likely the two main reasons contributing to the low internal consistency finding in the present study.

Similar to the problem in the internal consistency results, the factor loadings of the ICOPES-TW in the EFA were not high (ranging between 0.24 and 0.63). Nevertheless, most of the factor loadings were acceptable (i.e., > 0.3 or 0.4) [31, 41], with the exception of the visual subscale (0.24). This finding indicates that visual problems could be an independent problem from other problems (i.e., cognition, locomotion, and hearing). However, future studies are needed to explore potential underlying mechanisms to explain why visual problems do not correlate with other problems among older people.

Apart from low internal consistency, the ICOPES-TW was found to have satisfactory (and expected) associations with all external criteria, indicating its potential to be a good screening tool despite the low internal consistency. Higher ICOPES-TW scores, indicating poor intrinsic capacity, were significantly associated with poor ADL (including BADL and IADL), low QoL, higher frailty, older age, and low educational level. Because intrinsic capacity involves older people’s ability to function in daily activities (e.g., cognition, mobility, hearing, and visual ability) [8, 9, 14–16], older people are likely to have ADL problems when their intrinsic capacity is poor. Indeed, prior evidence shows that scores on other ICOPE instruments (in different forms from the ICOPES-TW used in the present study) were associated with poor BADL and IADL [9, 15]. Moreover, it was observed that the two ICOPES-TW domain scores were highly associated with each other and the entire ICOPES-TW score, while the entire ICOPES-TW score and its two domain scores were only moderately (or weakly) associated with scores on all the external measures (including age, BADL, IADL, QoL, and frailty). This indicates that the concepts assessed in the ICOPES-TW were different from these external variables, and supports the notion that intrinsic capacity is a different concept from age, ADL, QoL, and frailty [42].

When evaluating frailty of older people, they can be classified into three levels (i.e., robust, prefrailty, and frailty). The concept of intrinsic capacity also includes the ability of being robust among older people [9]. Therefore, it is unsurprising that the ICOPES-TW was associated with higher frailty and older age in the present study given that older age is associated with frailty [43]. Additionally, QoL was associated with score on the ICOPES-TW and this finding may be explained by the impaired ADL and severe level of frailty. That is, poorer intrinsic capacity may lead to poorer ADL and a more severe level of frailty [9, 15], and poorer ADL and frailty may in turn result in lower QoL [30, 36]. Moreover, with the use of CFS and BI scores, the ICOPES-TW was found to have good predictive validity (i.e., AUC > 0.7). The ICOPES-TW total score had the best precision, followed by the body functionality factor score and life adaptation score in predicting at-risk frailty and ADL dependency. However, the predictive validity needs to be treated with caution because the ICOPES-TW, CFS, and BI were collected simultaneously. The association between educational level and score on the ICOPES-TW may be explained by health literacy. More specifically, older people with higher levels of education are likely to have better health literacy [44], which may protect them from unhealthy behaviors [45] and maintain their intrinsic capacity.

There are some limitations in the present study. First, the present study did not carry out test-retest reliability and responsiveness of the ICOPES-TW. Therefore, it remains unclear if the ICOPES-TW has consistent measurement across time when older people have no changes in intrinsic capacity and if the ICOPES-TW can genuinely assess changes in intrinsic capacity. Future studies are therefore needed to explore the reproducibility and responsiveness of the ICOPES-TW for Taiwanese healthcare providers to have better confidence in using the ICOPES-TW in screening and program evaluations. Moreover, the present study was unable examine the predictability of the ICOPES-TW because of its cross-sectional design. Future studies are therefore needed to examine the predictability of the ICOPES-TW as a screening tool. Second, the data were collected using a convenience sampling design in Southern Taiwan. Therefore, the generalizability of the present findings is limited. Moreover, the numbers of participants in each of the three subsamples were imbalanced (i.e., the entire sample comprised a high proportion of relatively healthy individuals from community settings, and much lower proportions of participants recruited from outpatient clinics and inpatient wards). Therefore, most of the measures in the present study had ceiling effects. This can result in some biased distributions and could have impacted on the correlations between the ICOPES-TW and other external measures. Therefore, there is an issue of non-representativeness in the present sample mainly because the study used a convenience sample, which makes it very difficult to weight the results of the sample from the three population sources. Third, some measures in the present study were based on the nature of self-report. Therefore, social desirability bias (and other biases such as memory recall) may have occurred when the participants responded to some of the self-report measures. Lastly, the present study did not have any gold standard tool to examine the criterion-related validity of the ICOPES-TW. Therefore, future studies need to develop other psychometric tools so that criterion-related validity of the ICOPES-TW can be further examined.

## Conclusion

The present study suggests that the ICOPES-TW may be a useful screening tool for healthcare providers to quickly evaluate intrinsic capacity among Taiwanese older people. Although the subscales in the ICOPES-TW had lower internal consistency than desired, the summed score of the ICOPES-TW according to the WHO ICOPE framework plus the category of medication (added by the Taiwan government) was moderately associated with BADL, IADL, QoL, and frailty. Therefore, by screening with the ICOPES-TW, Taiwanese healthcare providers are likely able to identify older people who might have health problems or are at risk of frailty through the quick assessment of items related to body functionality and life adaptation. However, the present study only provided initial psychometric evidence regarding the ICOPES-TW, and further studies are needed to explore more deeply additional psychometric properties of the ICOPES-TW, such as sensitivity, specificity, reproducibility, and responsiveness.

## Data Availability

The datasets generated and/or analyzed during the present study are not publicly available owing to patient privacy and ethical issues. However, they are available from the corresponding author upon reasonable request.

## Abbreviations

WHO: World Health Organization
ICOPE: Integrated Care for Older People
ICOPES-TW: ICOPE screening tool for Taiwanese
QoL: Quality of life
BADL: Basic activity of daily living
IADL: Instrumental activity of daily living
MMSE: Mini Mental State Examination
MoCA: Montreal Cognitive Assessment
NCKUH: National Cheng Kung University Hospital
CFS: Clinical Frailty Scale
BI: Barthel Index
Lawton IADLS: Lawton Instrumental Activities of Daily Living Scale
EFA: Exploratory factor analysis
ANOVA: Analysis of variance
